# *Journal of Experimental & Clinical Cancer Research* reviewer acknowledgement 2015

**DOI:** 10.1186/s13046-016-0305-3

**Published:** 2016-02-05

**Authors:** Mauro Castelli

**Affiliations:** Regina Elena National Cancer Institute, Rome, Italy

## Abstract

The editors of *Journal of Experimental & Clinical Cancer Research* would like to thank all our reviewers who have contributed their time and expertise to the journal in Volume 34 (2015).

**Miguel Abal**

Spain

**Eliana Abdelhay**

Brazil

**Sandra Ackermann**

Germany

**Nitin Agarwal**

United States

**Ninna Aggerholm-Pedersen**

Denmark

**Fe Ahmed**

United States

**Yoshimitsu Akiyama**

Japan

**Hunaian Alam**

United States

**Stefano Alcaro**

Italy

**Eiman Aleem**

Sweden

**Irshad Ali**

United States

**Raquel Almeida**

Portugal

**Sudhakar Ammanamanchi**

United States

**Jie An**

United States

**Zoran Andric**

United States

**Cinzia Antognelli**

Italy

**Chiara Arienti**

Italy

**Maria Laura Avantaggiati**

United States

**Hideo Baba**

Japan

**Editta Baldini**

Italy

**F Baltazar**

Portugal

**Rameshwar Bamezai**

India

**Hans-Guenter Becker**

Germany

**Gautam Bedadala**

United States

**Barbara Belletti**

Italy

**M Beloueche-Babari**

United Kingdom

**Maria Serena Benassi**

Italy

**Felix Berlth**

Germany

**Bhaskar Bhattacharya**

Singapore

**Suvendra Bhattacharyya**

India

**Xiaofang Bian**

United States

**Rk Bikkavilli**

United States

**Annamaria Biroccio**

Italy

**Carmen Blanco-Aparicio**

Spain

**Giovanni Blandino**

Italy

**Camilla Böckelman**

Finland

**Ua Bommer**

Australia

**Benjamin Bonavida**

United States

**Mathias Bonmarin**

Switzerland

**Gianluca Bossi**

Italy

**Michael Bouchard**

United States

**Meriem Boukerroucha**

Belgium

**Corinne Bousquet**

France

**Emilio Bria**

Italy

**Nigel T Brockton**

Canada

**Alexander Brodsky**

United States

**Christian Bronner**

France

**Steven Brower**

United States

**Ingvild Johnsen Brusevold**

Norway

**Helen E Bryant**

United Kingdom

**Natalie Burrows**

United Kingdom

**Jo Caers**

Belguim

**Qing Song Cai**

United States

**Qingchun Cai**

United States

**Paolo Cajano**

United Kingdom

**Matteo Caleo**

Italy

**Lido Calorini**

Italy

**Ignacio Camacho-Arroyo**

Mexico

**Michele Caraglia**

Italy

**Valentina Casadio**

Italy

**Javier Castresana**

Spain

**V Catalano**

Italy

**Muge Celiktas**

United States

**K Chahed**

France

**Kishore Challagundla**

United States

**Pradeep Chaluvally Raghavan**

United States

**Colin Champ**

United States

**A Chatterjee**

India

**Naz Chaudary**

Canada

**Qi Chen**

United States

**Guobing Chen**

United States

**Zhe-Sheng Chen**

United States

**Y Chen**

China

**Qi Chen**

United States

**Eric Chevet**

France

**Ch Cho**

South Korea

**Salem Chouaib**

France

**Luiz Chuffa**

Brazil

**Mara Cirone**

Italy

**Bruno Clement**

France

**James Cody**

United States

**Helen M Coley**

United Kingdom

**Bl Coomber**

Canada

**Paola Costelli**

Italy

**Jack B Cowland**

Denmark

**Marco Crescenzi**

Italy

**Monika Csoka**

Hungary

**Kevin Cullen**

United States

**Bingbing Dai**

United States

**Marina De Bernard**

Italy

**Francesca De Lorenzi**

Italy

**Juan R. De Los Toyos**

Spain

**Deilson Elgui De Oliveira**

Brazil

**Marina De Rosa**

Italy

**Sharon Demorrow**

United States

**Wolfram Dempke**

United Kingdom

**Christèle Desbois-Mouthon**

France

**Harshil Dhruv**

United States

**Silvia Di Agostino**

Italy

**Giorgio Di Matteo**

Italy

**Francesca Di Modugno**

Italy

**La Di Pietro**

United States

**Giuliana Di Rocco**

Italy

**Alberto Di Ronza**

United States

**Deborah Dillon**

United States

**Liya Ding**

United States

**Husheng Ding**

United States

**Shareen Doak**

United Kingdom

**Lanfeng Dong**

Australia

**Hu Dong**

United States

**Jessica Donington**

United States

**Gabriella D'Orazi**

Italy

**Kristina Drott**

Sweden

**Xiaoli Du**

United States

**Diana Dudziak**

Germany

**Randy Dull**

United States

**Bilikere S Dwarakanath**

India

**Boban Erovic**

Austria

**J Esteve**

Spain

**Thomas Fahey**

United States

**Qipeng Fan**

United States

**Zhichao Fan**

China

**Huaqiang Fang**

United States

**A Farazmand**

Iran

**Matteo Fassan**

Italy

**Lorenzo Federico**

United States

**Armando Felsani**

Italy

**Cristiano Ferlini**

United States

**Barbara Fingleton**

United States

**Matthias Fischer**

Germany

**Paul Fisher**

United States

**M Flint**

United Kingdom

**Tullio Florio**

Italy

**Giulia Fontemaggi**

Italy

**Fl Forti**

Brazil

**Orazio Fortunato**

Italy

**Florentia Fostira**

Greece

**Heather Francis**

United States

**Philippe G Frank**

France

**Paula Fresco**

Portugal

**Terence Friedlander**

United States

**Eleonore Fröhlich**

Austria

**Ezequiel M Fuentes-Panana**

Mexico

**Naotake Funamizu**

Japan

**Eduard Gabrielson**

United States

**Sehamuddin Galadari**

United Arab Emirates

**Rossella Galati**

Italy

**Jt Gao**

Taiwan, Republic Of China

**Chenxi Gao**

United States

**Manoj Garg**

Singapore

**Manuela Gariboldi**

Italy

**Sylvie Gazzeri**

France

**Sk Ghosh**

United States

**Patrizio Giacomini**

Italy

**Antonio Giordano**

United States

**Paola Giussani**

Italy

**Gabriel Glockzin**

Germany

**Fernand Gobeil**

Canada

**Nicolas Goffart**

Belgium

**Daniel Goldenberg**

Israel

**Lauren Gollahon**

United States

**Aliesha Gonzalez-Arenas**

Mexico

**Ramakrishnan Gopalakrishnan**

United States

**Te Goranova**

Bulgaria

**Christophe Grosset**

France

**Ludmila Grzybowska-Szatkowska**

Poland

**Z Gu**

United States

**Sushma Reddy Gundala**

United States

**Ailin Guo**

United States

**Linlin Guo**

Canada

**Rui Guo**

China

**Ailin Guo**

United States

**Hou-Fu Guo**

United States

**F Guo**

China

**Amor Hajri**

Germany

**Petra Hamerlik**

Denmark

**Bangmin Han**

China

**Sm Hanash**

United States

**Swei Sunny Hann**

China

**Torsten Hansen**

Germany

**Jeffrey K Harrison**

United States

**Mitsutoki Hatta**

Japan

**Shoichi Hazama**

Japan

**Jin-Shen He**

China

**Christopher Heeschen**

Spain

**M Herreros-Villanueva**

Spain

**Reginald Hill**

United States

**Karsten Hiller**

Luxembourg

**Huub Hoefsloot**

Netherlands

**Lihong Huo**

United States

**H Ide**

Japan

**Subburaj Ilangumaran**

Canada

**Landon Inge**

United States

**Juan Iovanna**

France

**Mohd Askandar Iqbal**

United States

**T Iwaya**

Japan

**Margit Janát-Amsbury**

United States

**Miguel Japon**

Spain

**Ali R Jazirehi**

United States

**Bipulendu Jena**

United States

**Ping Ji**

United States

**Zhou Jian**

China

**Xinguo Jiang**

United States

**Chengcheng Jin**

United States

**Lei Jin**

Australia

**Chengcheng Jin**

United States

**Lei Jin**

Australia

**Siwanon Jirawatnotai**

Thailand

**Shweta Joshi**

United States

**Claire Josse**

Belgium

**Eva Juengel**

Germany

**Ma Jun**

China

**Klaus Jung**

Germany

**Kerstin Junker**

Germany

**Guillermo Juvenal**

Argentina

**Paul Kachel**

Germany

**F Kalalinia**

Iran

**Steve Kalloger**

Canada

**Charis Kalogirou**

Germany

**Hirofumi Kamachi**

Japan

**Rupinder Kanwar**

Australia

**Yh Kao**

Taiwan

**Sid Kar**

United States

**Vasilios Karavasilis**

Greece

**Peeter Karihtala**

Finland

**M Katoh**

Japan

**X Ke**

China

**Bastian Keck**

Germany

**Eric Kelley**

United States

**Syed Khundmiri**

United States

**Michaela Kirschner**

Switzerland

**Uwe Klinge**

Germany

**Roman Kloeckner**

Germany

**Bruno Koehler**

Germany

**Dorde Komljenovic**

Germany

**Manousos Konstadoulakis**

Greece

**Udo Kontny**

Germany

**Matthias Kroiss**

Germany

**Bernd Krone**

Germany

**Jerome Kroonen**

Belgium

**Sebastian Krug**

Germany

**Rajiv Kumar**

Germany

**Bekir Kuru**

Turkey

**Marie-Christine Kyrtsonis**

Greece

**Ma La Barge**

United States

**Silvia La Porta**

Germany

**Shruti Lal**

United States

**Paula Yp Lam**

Singapore

**John Lamar**

United States

**Jiang Lan**

United States

**Sven Lang**

Germany

**Christer Larsson**

Sweden

**Amanda C Larue**

United States

**Konstantinos Lasithiotakis**

Greece

**Mark Lawler**

United Kingdom

**Jasmine G Lee**

United States

**You Mie Lee**

South Korea

**Ulrich Lehmann**

Germany

**Carlo Leonetti**

Italy

**Chao Li**

United States

**Shaoguang Li**

United States

**Chao Li**

United States

**Rui Li**

United States

**Fengzhi Li**

United States

**Jing Li**

United States

**Chao Li**

United States

**Jiang Li**

China

**Rui Li**

United States

**Z Li**

China

**Yihao Li**

Netherlands

**Jun Li**

United States

**Long-Cheng Li**

United States

**Yu-Chih Liang**

Taiwan

**Shu-Chun Lin**

Taiwan

**Alexander Link**

Germany

**D Liu**

Armenia

**Gang Liu**

United States

**Peng Liu**

United States

**Bingya Liu**

China

**Zhixia Liu**

United States

**Lucia Lopalco**

Italy

**Andrew Lowy**

United States

**Yong Lu**

United States

**Wei Lu**

China

**John M Luk**

China

**Yz Luo**

China

**Leandro Luongo De Matos**

Brazil

**Li Ma**

United States

**Kyle Macbeth**

United States

**Marthandan Mahalingam**

United States

**Sunipa Majumdar**

United States

**Peter Malfertheiner**

Germany

**Pp Manna**

India

**Rohit Mathur**

United States

**Mahide Matsuda**

Japan

**Marion Maurel**

Ireland

**Demetra Mavri-Damelin**

South Africa

**F Mehrkhani**

United States

**Andre Mencalha**

Brazil

**Raul Mendez**

Spain

**Anna Maria Mileo**

Italy

**Vedrana Milinkovic**

Germany

**Js Miller**

United States

**Prasun J Mishra**

United States

**Abhisek Mitra**

United States

**Yoichi Mizutani**

Japan

**Holger Moch**

Switzerland

**Jaime F Modiano**

United States

**Ananda Mooker**

United States

**Claire Morgan**

United Kingdom

**Massimo Moro**

Italy

**Marcella Mottolese**

Italy

**Caroline Moyret-Lalle**

France

**Ping Mu**

China

**Shigeyuki Murono**

Japan

**Mohd Rais Mustafa**

Malaysia

**H Nakano**

Japan

**Shoji Natsugoe**

Japan

**Mitsuteru Natsuizaka**

Japan

**Roman Nawroth**

Germany

**Ma Nelson**

United States

**Ruifang Niu**

China

**H Ohtsuka**

Japan

**Masato Okamoto**

Japan

**Riki Okita**

Japan

**Hiroshi Okumura**

Japan

**Takuji Okusaka**

Japan

**Pd Ottewell**

United Kingdom

**M Oudin**

United States

**Adriana Paes Leme**

Brazil

**Marco Paggi**

Italy

**Jay Palem**

United States

**Claudia Palena**

United States

**Philip Palmbos**

United States

**Jaroslaw Paluszczak**

Poland

**Wen Pan**

China

**Jianji Pan**

China

**Yunyan Pan**

China

**David Panka**

United States

**Athanasios G. Papavassiliou**

Greece

**Monika Papiez**

Poland

**Suman Paritosh**

United States

**Anna Paszel-Jaworska**

Poland

**Naeem K Patil**

United States

**Prakash Paudyal**

United States

**Laura Pazzaglia**

Italy

**Xt Pei**

China

**Sravan Penchala**

United States

**Haiyong Peng**

United States

**Qian Pengxu**

United States

**Christine Perret**

France

**S Persad**

Canada

**Deepak Perumal**

United States

**Jm Peula-Garcia**

Spain

**Giulia Piaggio**

Italy

**Nicola Picardi**

Italy

**Raffaella Pippa**

United Kingdom

**Nabendu Pore**

United States

**Alessandro Porrello**

Italy

**Kartick Pramanik**

United States

**Jochen H.M. Prehn**

Ireland

**Carlo Pucillo**

Italy

**David Puett**

United States

**Hui Qian**

China

**Bing Qiu**

China

**Lianghu Qu**

China

**Zhiwei Quan**

China

**Eveline Queiroz**

Brazil

**Sandeep Rajput**

United States

**Sheela Ramanathan**

Canada

**Annamari Ranki**

Finland

**Diane Renz**

Germany

**Mario Ricciardi**

Italy

**Lucia Ricci-Vitiani**

Italy

**Sara Richter**

Italy

**Jochen Roessler**

Germany

**Laura Rosanò**

Italy

**Anja Rudolph**

Germany

**Antonio Russo**

Italy

**Jolanta Saczko**

Poland

**Ahmad R Safa**

United States

**H Sakai**

Japan

**Frederic Saltel**

France

**Erica Salvati**

Italy

**Belinda Sanchez**

Cuba

**Gustavo Santa Cruz**

Argentina

**Daniele Santini**

Italy

**Webster Santos**

United States

**Alv Savio**

Brazil

**Orazio Schillaci**

Italy

**Stefanie Schrauwen**

Belgium

**Hildegard Schuller**

United States

**Andreas Scorilas**

Greece

**Archana Sehrawat**

United States

**Elena Seviour**

United States

**Na Shang**

United States

**Bal Krishan Sharma**

India

**Sherven Sharma**

United States

**Bal Krishan Sharma**

India

**Anuraag Shrivastav**

Canada

**Sanjeev Shukla**

United States

**Shirish Shukla**

United States

**Nyati Shyam**

United States

**Bhuminder Singh**

United States

**Garima Singhal**

United States

**Doris Siwak**

United States

**Tania Slatter**

New Zealand

**Alex Soltermann**

Switzerland

**Philippe Soubeyran**

France

**Roland Stauber**

Germany

**Joachim Steinbach**

Germany

**Sabrina Strano**

Italy

**Eva Wessel Stratford**

Norway

**Rongjian Su**

China

**Meng Su**

United States

**Subbaya Subramanian**

United States

**Naoko Sueoka-Aragane**

Japan

**Haruhiko Sugimura**

Japan

**Sangeetha Sukumari-Ramesh**

United States

**Ping Sun**

United States

**Sheng Sun**

United States

**Haibo Sun**

United States

**Zhenhe Suo**

Norway

**Yasuyuki Suzuki**

Japan

**A Takahashi**

Japan

**Wei Tang**

United States

**Rong-Hua Tao**

United States

**Atsushi Tatsuguchi**

Japan

**Helge Taubert**

Germany

**Emma Tham**

Sweden

**Rabi Thangaiyan**

Greece

**Hamsa Thayele Purayil**

United States

**Faisal Thayyullathil**

United Arab Emirates

**James Thorne**

United Kingdom

**Xiaohong Tian**

China

**Tilman Todenhofer**

Germany

**M Tomasetti**

Italy

**Maria Lauda Tomasi**

United States

**Patrizia Tosi**

Italy

**Ben Tran**

Australia

**Pankaj Trivedi**

Italy

**Maria Fernanda Troncoso**

Argentina

**Koji Tsuboi**

Japan

**Ryouichi Tsunedomi**

Japan

**Guldeep Uppal**

United States

**Anni Vedeler**

Normway

**Thirunavukkarasu Velusamy**

United States

**Patrick Venables**

United Kingdom

**Maryna Veremieva**

Ukraine

**Emmy Verschuren**

Finland

**Marcal Vilar**

Spain

**Bruno Vincenzi**

Italy

**M Vinken**

Belgium

**Sukesh Voruganti**

India

**Demitrios Vynios**

Greece

**Renu Wadhwa**

Japan

**Kepeng Wang**

United States

**Yubao Wang**

United States

**Wenbo Wang**

China

**Yingying Wang**

China

**Ying Wang**

United States

**S Wang**

China

**Andrea Wang-Gillam**

United States

**Wei Wei**

United States

**Yaguang Weng**

China

**Kai Wermker**

Germany

**Richard White**

United States

**Joanna Wietrzyk**

Poland

**Werner Windischhofer**

Austria

**Chi-Huey Wong**

Taiwan

**Chih-Ching Wu**

Taiwan

**Wx Wu**

China

**Minghui Wu**

United States

**Qianghua Xia**

China

**Badrul Yahaya**

Malaysia

**Kimihiro Yamashita**

Japan

**Max Yan**

Australia

**Shun-Fa Yang**

Taiwan

**Huilin Yang**

China

**Chia-Yu Yang**

Taiwan

**Huilin Yang**

China

**Lifang Yang**

China

**Xingsheng Yang**

China

**Clayton Yates**

United States

**Zhong Ye**

United States

**Bin Yi**

United States

**Qi Yin**

United States

**Takeito Yokobori**

Japan

**Gs Yoon**

South Korea

**Go Yoshida**

Japan

**Takayuki Yoshino**

Japan

**Xiaofei Yu**

United States

**Flora Zagouri**

United Kingdom

**Andrew Cw Zannettino**

Australia

**Kostas Zarogoulidis**

Greece

**Hong-Tao Zhang**

China

**Mei Zhang**

United States

**Yaqing Zhang**

United States

**Shiwu Zhang**

China

**Yu Zhang**

United States

**Jing Zhang**

United States

**Yaqing Zhang**

United States

**Zhengmao Zhang**

United States

**Kun Zhang**

United States

**Xi Zhang**

United States

**L Zhang**

China

**Yaqing Zhang**

United States

**Jun Zhao**

China

**Yuhua Zhao**

China

**Ming Zhao**

United States

**Wei Zhao**

United States

**Chenghai Zhao**

China

**Jian Zhong**

United States

**Weihua Zhou**

United States

**Valentina Zuco**

Italy

